# Analyzing Complex Problem Solving by Dynamic Brain Networks

**DOI:** 10.3389/fninf.2021.670052

**Published:** 2021-12-10

**Authors:** Abdullah Alchihabi, Omer Ekmekci, Baran B. Kivilcim, Sharlene D. Newman, Fatos T. Yarman Vural

**Affiliations:** ^1^Department of Computer Engineering, Middle East Technical University, Ankara, Turkey; ^2^Department of Psychological and Brain Sciences, Indiana University, Bloomington, IN, United States

**Keywords:** fMRI, machine learning, brain networks, tower of London (TOL), complex problem solving

## Abstract

Complex problem solving is a high level cognitive task of the human brain, which has been studied over the last decade. Tower of London (TOL) is a game that has been widely used to study complex problem solving. In this paper, we aim to explore the underlying cognitive network structure among anatomical regions of complex problem solving and its subtasks, namely *planning* and *execution*. A new computational model for estimating a brain network at each time instant of fMRI recordings is proposed. The suggested method models the brain network as an Artificial Neural Network, where the weights correspond to the relationships among the brain anatomic regions. The first step of the model is preprocessing that manages to decrease the spatial redundancy while increasing the temporal resolution of the fMRI recordings. Then, dynamic brain networks are estimated using the preprocessed fMRI signal to train the Artificial Neural Network. The properties of the estimated brain networks are studied in order to identify regions of interest, such as hubs and subgroups of densely connected brain regions. The representation power of the suggested brain network is shown by decoding the planning and execution subtasks of complex problem solving. Our findings are consistent with the previous results of experimental psychology. Furthermore, it is observed that there are more hubs during the planning phase compared to the execution phase, and the clusters are more strongly connected during planning compared to execution.

## 1. Introduction

Complex problem solving is a very crucial ability of the human brain, which covers a large number of high-level cognitive processes, including strategy formation, coordination, sequencing of mental functions, and holding information online. These complex high-level cognitive processes make the inner workings of problem solving a challenging task.

**Figure 1:**
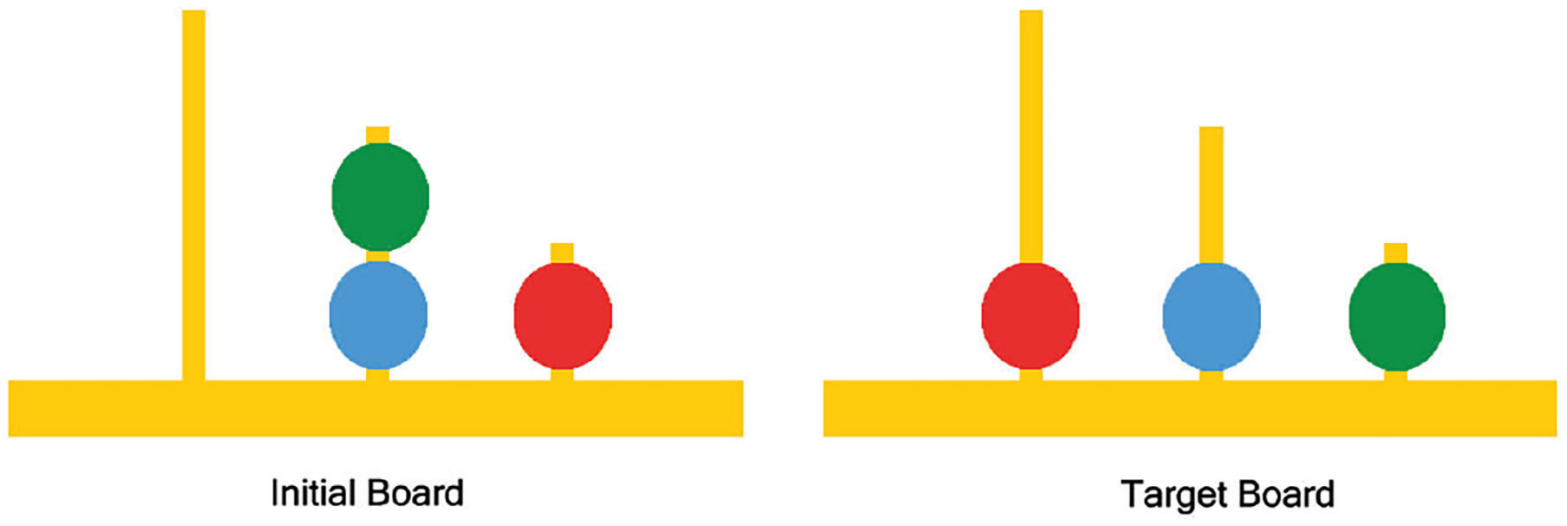
Example tower of London (TOL) puzzle.

The standard method for neuro-analysis of complex problem solving in the literature is to study the fMRI data recorded while the subjects play the Tower of London (TOL) game, designed by Shallice (1982). TOL game consists of three bins having different capacities with colored balls placed in the bins; the aim is to rearrange the balls from their initial state to a predetermined goal state while moving one ball at a time and taking into consideration the limited capacity of each bin (as shown in Figure 1).

TOL game has been primarily employed to study the effect of various properties of complex problem solving performance in healthy subjects. The predictive power of working memory, inhibition, and fluid intelligence on TOL performance has been investigated with consideration of factors such as age, gender, exercise, etc. (Unterrainer et al., 2004, 2005; Zook et al., 2004, 2006; Boghi et al., 2006; Albert and Steinberg, 2011; Chang et al., 2011; Desco et al., 2011; Kaller et al., 2012). Additionally, TOL has been used to investigate the effect of various clinical disorders on functions associated with the prefrontal cortex such as planning. For example, the task has been utilized in neuroimaging studies to identify executive dysfunction by examining differential cognitive activation patterns in people suffering from neurological disorders like epilepsy, seizures, depression, Parkinson's and schizophrenia (Goethals et al., 2005; Rasser et al., 2005; Rektorova et al., 2008; MacAllister et al., 2012).

The classic work of Newell and Simon (Newell et al., 1957; Simon and Newell, 1971) hypothesized three distinct phases of complex problem solving: construction of problem representation, elaboration to search for operators to solve the problem, and execution to implement the solution. Despite being a well respected theory, there is little to no evidence from cognitive neuroimaging that supports this hypothesis directly. Consequently, refinements of the theory, such as online planning in which elaboration and execution phases are interspersed, and mechanisms such as schema development that may suggest qualitative differences between good and poor problem-solvers, are understood even less. The primary reason for this state of affairs is that cognitive neuroimaging in general and fMRI analysis, in particular, tends to ignore the temporal aspect of how brain activation and network connectivity evolve during complex cognitive tasks. Further, much of the existing methods have tended to test theoretical cognitive models by searching for brain data that fit those models, rather than using the brain data themselves to inform us about cognition.

Numerous studies have proposed various computational models in order to build brain networks from fMRI measurements, both during cognitive tasks or during resting state. These studies represent a shift in the literature toward brain decoding algorithms that are based on the connectivity patterns in the brain motivated by the findings that these patterns provide more information about cognitive tasks than the isolated behavior of individual groups of voxels or anatomical regions (Lindquist, 2008; Ekman et al., 2012; Shirer et al., 2012; Richiardi et al., 2013; Onal et al., 2017). Some of these studies focused on the pairwise relationships between voxels or brain regions. For example, Pearson correlation has been used in order to construct undirected functional connectivity graphs at different frequency resolutions in Richiardi et al. (2011). Also, pairwise correlations and mutual information have been used in order to build functional brain networks in various studies aiming to investigate the network differences between patients with Schizophrenia or Alzheimer's disease and healthy subjects (Lynall et al., 2010; Menon, 2011; Kurmukov et al., 2017). Others used partial correlation along with constrained linear regression to generate brain networks in Lee et al. (2011).

In our previous studies, we take advantage of the locality property of the brain by constructing local mesh networks around each brain region. Then, we represent the entire brain network as an ensemble of local meshes. In these studies, we estimated the Blood-Oxygenation Level Dependent (BOLD) response of each brain region as a linear combination of the responses of its "closest" neighboring regions. Then, we solved the systems of linear equations using various regression techniques. Our team applied Levinson-Durbin recursion in order to estimate the edge weights of each local star mesh, where the nodes are the neighboring regions of the seed brain region (Fırat et al., 2013; Alchihabi et al., 2018). We also used ridge regression to estimate edge weights while constructing the local mesh networks across windows of time series of fMRI recordings (Onal et al., 2015, 2017).

In this study, we present a novel approach for estimating dynamic brain networks, which represent the relationship among the brain anatomic regions at each time instant of the fMRI recordings. The approach models the relationship among the anatomical brain regions as an Artificial Neural Network (ANN), where the edge weights correspond to the arc weights of the brain network. The idea of modeling the brain network as an ANN is first introduced in our lab (Kivilcim et al., 2018), where the model can be constructed to estimate both directed and undirected brain graphs. In this study, we further extend this idea to estimate dynamic brain networks. We also explore the validity and representation power of the suggested brain network by analyzing its statistical properties using the methods suggested in Bassett and Bullmore (2006), Power et al. (2010), Rubinov and Sporns (2010), and Park and Friston (2013).

Several network measures, such as measures of centrality, which identify potential hubs and measures of functional segregation, which detect densely interconnected clusters of nodes, provide means to analyze both individual components of brain network and the brain network as a whole. As a result, these network measures reveal and characterize various aspects of inter-dynamics of brain regions enabling us to analyze and compare different brain network snapshots. The properties of the dynamic brain networks are studied in order to identify the active anatomical regions during both planning and execution phases of complex problem solving. Potential hubs and clusters of densely connected brain regions are identified for both subtasks. Furthermore, the distinctions and similarities between planning and execution networks are highlighted. The results identify both active and inactive hub regions as well as clusters of densely connected anatomical regions during complex problem-solving. In addition, results show that there are more potential hubs during the planning phase compared to the execution phase. Also, the clusters of densely interconnected regions are significantly more strongly connected during planning compared to execution. Finally, we studied the decoding power of the suggested brain network model by using simple machine learning methods to classify two phases of complex problem solving, namely, planning and execution.

## 2. TOL Experiment Procedure

In this section, we introduce the details of the experiment as well as data collection and preprocessing methods.

### 2.1. Participants and Stimuli

18 college students aged between 19 and 38 participated in the experiment after signing informed, written consent documents approved by the Indiana University Institutional Review Board. The subjects solved a computerized version of TOL problem; two configurations were presented at the beginning of each puzzle: the initial state and the goal state. The subjects were asked to transform the initial state into the goal state using the minimum number of moves. However, the subjects were not informed of the minimum number of moves needed to solve a given puzzle nor of the existence of multiple solution paths.

### 2.2. Procedure

Each subject underwent a practice session before entering the scanning session to acquaint subjects with the TOL problem. The subjects were given the following instructions: "You will be asked to solve a series of puzzles. The goal of the puzzle is to make the 'start' or 'current' state match the 'goal' state (They were shown an example). Try to solve the problems in the minimum number of moves by planning ahead. Work as quickly and accurately as possible, but accuracy is more important than speed."

The scanning session consisted of 4 runs, each run included 18 timed puzzles, with a 5-s planning only time slot during which subjects were not allowed to move the balls. However, they were allowed to continue planning after the 5 s planning only time slot if they chose to do so. Following every puzzle, there was a 12-s rest period where subjects focused on a plus sign in the center of the screen. Each run was also followed by a 28-s fixation period.

**Figure 2:**
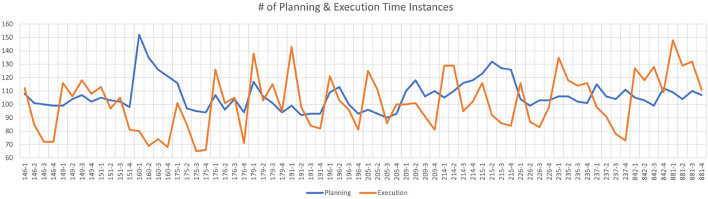
The number of planning and execution instances for each run of all subjects. Horizontal axis indicates the subject ID for each run (there are total of 18 subject x4 runs = 72 runs), whereas the vertical axis shows the number of time instances for planning (blue) and execution (orange) per run.

The planning task is defined from the start of the puzzle until the subject's first move. The execution task is defined from the subject's first move until the end of the puzzle. During the experiments, both planning and execution times change across the subjects, runs and puzzles. The average planning time instances per puzzle is 5.91 and the average time instances for execution per puzzle is 5.63 over all puzzles and subjects. The average total planning time instances per run is 106.35 and the average total execution time instances per run is 101.28 over all subjects. Figure 2 shows the total number of planning and execution instances for each run of all subjects. Each mark on the horizontal axis refers to a given run of a specific subject. As it can be observed from Figure 2, the data is quite balanced between the planning and execution phases (average of 101 planning and 106 execution across the subjects per each run). For this reason, we did not augment the classes or eliminate some samples in the dataset for balancing the classes. The details of the dataset are summarized in Table 1.

**Table 1:** Summary of TOL dataset.

# of Subjects	18
# of Runs/subject	4
# of Puzzles/run	18
Avg. Planning instances/puzzle	5.91
Avg. Execution instances/puzzle	5.63
Avg. Planning instances/run	106.35
Avg. Execution instances/run	101.28
Total planning instances for 18 x 4 runs	7,657
Total execution instances for 18 x 4 runs	7,292

*Note that the planning instances (7657) and execution instances (7292) are quite balanced*.

### 2.3. fMRI Data Acquisition and Preliminary Analysis

The fMRI images were collected using a 3 T Siemens TRIO scanner with an 8-channel radio frequency coil located in the Imaging Research Facility at Indiana University. The images were acquired in 18 5 mm thick oblique axial slices using the following set of parameters: TR = 1,000 ms, TE = 25 ms, flip angle = 60°, voxel size = 3.125 mm × 3.125 mm × 5 mm with a 1 mm gap.

The statistical parametric mapping toolbox was used to perform the preliminary data analysis that included: image correction for slice acquisition timing, resampling, spatial smoothing, motion correction and normalization to the Montreal Neurological Institute (MNI) EPI template. Further details concerning the procedure and data acquisition can be found in Newman et al. (2009) as we use the same data/participants in this study. It is also worth noting that we perform our analysis on all recorded puzzles, not only correctly solved ones, given that the aim of this study is to investigate the planning and execution networks in general. In future work, we aim to study the differences in the planning and execution networks between good problem-solvers and bad problem-solvers; in that case, we will make the distinction between correctly solved puzzles and unsolved puzzles. Furthermore, the entirety of our analysis is performed on the raw fMRI recordings; no first-level modeling or regressors are applied; rather, we use the recorded time series as our raw BOLD response.

**Figure 3:**
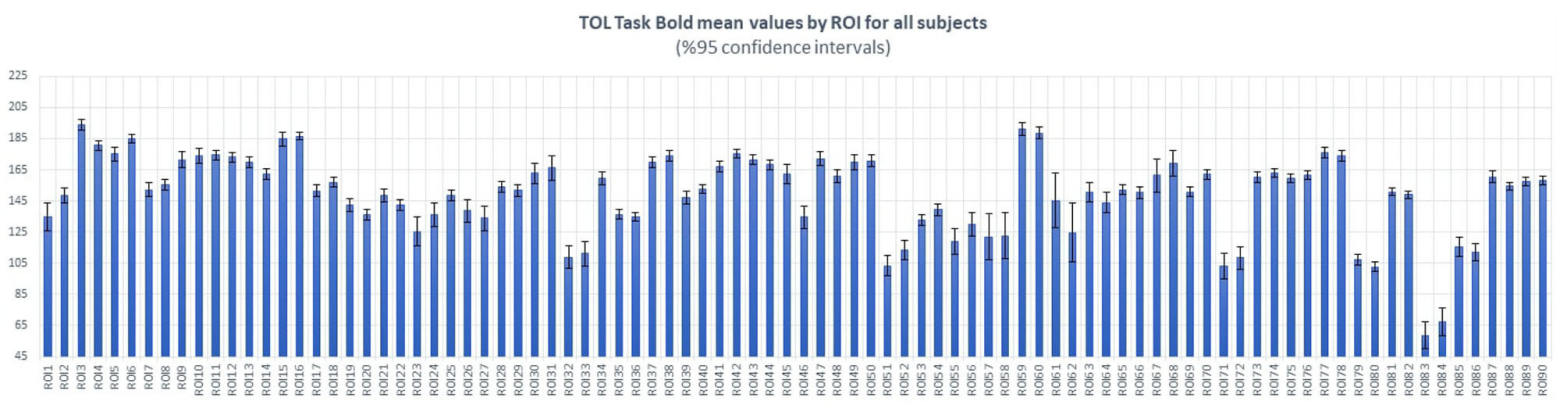
Estimated mean values and variations of BOLD activation of each brain anatomic region, over 18 subjects. Horizontal axis shows the index of anatomic regions, whereas the vertical axis shows the mean values (blue bars) and variances (black bars) of measured BOLD activation.

In order to investigate the inter-subject variability, we estimate the mean values and variance of BOLD activation of each brain anatomic region across all subjects. Figure 3 clearly shows the relatively low variations of the BOLD activation around the mean values of brain anatomic regions across 18 subjects.

## 3. Modeling Dynamic Brain Network as an Artificial Neural Network

Can we model the relationship among the anatomic regions as an Artificial Neural Network? If so, what is the validity and representation power of this network to analyze cognitive tasks such as complex problem solving? In this section, we suggest a computational model to represent the complex problem solving task as a dynamic brain network. In the next section, we shall explore the validity of this network and try to analyze the complex problem solving task of the human brain.

### 3.1. Preprocessing of the fMRI Recordings

In order to be able to estimate a dynamic brain network among the anatomic regions, we need to process the raw fMRI data for

- representation of anatomic regions,
- interpolation in time,
- injecting additive noise,

as explained below.

#### 3.1.1. Representation of Anatomic Regions

Each anatomic region is represented by a time series using voxel selection and averaging methods. Voxel selection reduces the dimension of fMRI data (185,000 voxels per brain volume) and eliminates the irrelevant voxels that do not contribute to the underlying cognitive process. ANOVA method is used to choose the most discriminative voxels and to discard the remaining ones (Cox and Savoy, 2003; Pereira et al., 2009; Afrasiyabi et al., 2016). The *f*-value score of each voxel ***v***_***i***_ is calculated from Equation (1):


(1) $$\begin{array}{l} f\text{\_}score_{i}=\frac{MSB\left(v_{i},y_{label}\right)}{MSW\left(v_{i},y_{label}\right)}, \end{array}$$

where ***y***_***label***_ is the label indicating the subtask (Planning or Execution). *MSB*(***v***_***i***_, ***y***_***label***_) is the mean square value between raw measured BOLD response of voxel *i* and the label vector ***y***_***label***_, which is calculated by Equation (2):


(2) $$\begin{array}{l} MSB\left(v_{i},y_{label}\right)=\frac{SSB\left(v_{i},y_{label}\right)}{df_{between}}, \end{array}$$

*SSB*(***v***_***i***_, ***y***_***label***_) is the sum of squares between ***y***_***label***_ and ***v***_***i***_, *df*_*between*_ is the number of groups minus one. *MSW*(***v***_***i***_, ***y***_***label***_) is the mean square value within voxel *i* and the label vector ***y***_***label***_ and it is calculated by Equation (3):


(3) $$\begin{array}{l} MSW\left(v_{i},y_{label}\right)=\frac{SSW\left(v_{i},y_{label}\right)}{df_{within}}, \end{array}$$

where *SSW*(***v***_***i***_, ***y***_***label***_) is the sum of squares within group and *df*_*within*_ is the degree of freedom within (total number of elements in ***v***_***i***_ and ***y***_***label***_ minus the number of groups).

We order the voxels according to their *f*-value scores. Then, the distribution of *f*-value scores of all voxels is plotted in order to determine the number of voxels to retain. Voxel selection is applied to the voxels of all brain regions except the ones located in the cerebellum, which we exclude during network extraction.

Each anatomic region is represented by averaging the BOLD response of the selected voxels, which resides in that region defined by automated anatomical labeling (AAL) atlas (Tzourio-Mazoyer et al., 2002) as shown in Equation (4):


(4) $$\begin{array}{l} r_{j}=\frac{\sum_{i\in \zeta \left[j\right]}v_{i}}{\left|\zeta \left[j\right]\right|} \end{array}$$

where ***r***_***j***_ is the representative BOLD response of region *j*, ***v***_***i***_ is the raw measured BOLD response of voxel *i* and ζ[*j*] is the set of selected voxels located in region *j*. The representative BOLD responses, ***r***_***j***_, enables us to investigate the role and contribution of each region to the planning and execution phases of the problem solving task.

#### 3.1.2. Interpolation

It is well-known that despite its high spatial resolution, fMRI signal has very low temporal resolution compared to EEG signal. In this study, we interpolate the fMRI signal in order to compensate for this drawback and study the effect of interpolation for estimating the brain networks and on decoding the planning and execution phases of TOL game.

In the TOL study, subjects solved a puzzle in at most 15 s and the sampling rate, TR, is 1,000 ms. Interpolation is used to increase the temporal resolution by estimating *z* extra brain volumes between each two consecutive measured brain volumes. As a result, the total number of available brain volumes for each puzzle becomes *n* + *z* * (*n* − 1), where *n* is the number of measured brain volumes of a given puzzle. We use the cubic spline interpolation function rather than linear interpolation methods in order to prevent edge effects and smoothing out the spikes between the measured brain volumes (McKinley and Levine, 1998).

In order to analyze the effect of time interpolation and to estimate an acceptable number of inserted brain volumes *z*, we compare the Fourier Transform of the fMRI signal computed before and after interpolation so that the frequency content of the signal is not distorted by interpolation. The original single-sided amplitude of the signal and the one obtained after interpolation are compared in order to ensure that interpolation is preserving the smooth peaks of the data in the frequency domain (Cochran et al., 1967; Frigo and Johnson, 1998).

#### 3.1.3. Injecting Gaussian Noise

When modeling a deterministic signal by a probabilistic method, adding noise to the signal decreases the estimation error in most of the practical applications. The final phase of preprocessing is adding Gaussian noise to the interpolated time series of the BOLD response in each anatomical region. For this purpose, instead of just injecting white noise, a rather informed noise, *colorful Gaussian noise*, is added. In order to reflect the corresponding brain region's properties, for each sample, the additive noise sample is generated from a Gaussian distribution having mean and variance of that anatomical region. These newly generated samples not only act like a natural regularizer to improve the generalization performance of brain decoding but also help making the Artificial Neural Network more stable when estimating the edge weights of the brain networks (Matsuoka, 1992; Reed et al., 1992).

Given a representative time series from a particular brain region, *i* represents the index of an anatomical region. The new samples are generated with vector addition of noise while preserving the signal-to-noise ratio (SNR) as in ${\tilde{r}}_{j}=r_{j}+\tau_{j}$, where **τ_*j*_** is a noise vector sampled from $N\left(\alpha_{noise}\mu \left(r_{j}\right),\beta_{noise}\sigma^{2}\left(r_{j}\right)\right)$, α_*noise*_ and β_*noise*_ are the scaling factors which are set empirically, to optimize the decoding performance.

### 3.2. Building Dynamic Brain Networks With Artificial Neural Networks

The above preprocessing methods yield a relatively high temporal resolution and smooth time series for each anatomic region compared to the row fMRI recordings.

In this section, we use the output of the preseprocessing step to estimate the relationship among the time series of anatomic regions at each time instance to generate a dynamic brain network, where the arc weights vary with respect to time instances.

#### 3.2.1. Partitioning the Time Series Into Fixed Size Internals and Defining the Brain Network for Each Window

As the first step, we partition each time series, which represents an anatomical region, into fixed-size windows. Each window, *win*(*t*), is centered at the measured brain volume at time instance, *t*. The size of each window is *Win*_*Size* = *z* + 1 brain volumes, where *z* is the number of interpolated brain volumes in each window. Equation (5) shows the time instances included in each window.


(5) $$\begin{array}{l} win(t)=\left[t-\left\lfloor \frac{z}{2}\right\rfloor ,\ldots ,t,\ldots ,t+\left\lceil \frac{z}{2}\right\rceil \right] \end{array}$$

We define a dynamic brain network, *N*(*t*) = (*V, W*(*t*)), for each time window *win*(*t*), where *V* is the set of nodes of the graph corresponding to the brain anatomical regions and *W*(*t*) = {*w*_*t,j,i*_|∀*i, j* ∈ *V*} is the directed weighted edges between the nodes of the graph within time window *win*(*t*). The nodes of the graph represent the AAL-defined brain regions (Tzourio-Mazoyer et al., 2002), except for the regions located in the cerebellum. The nodes are then pruned using voxel selection, as some anatomical regions contribute no voxels at all and get deleted from the set of nodes of the graph *V*.

Note that our aim is to label the BOLD responses measured at each brain volume as it belongs to one of the two phases of complex problem solving, namely, *planning and execution*. For this purpose, we represent each brain volume measured at a time instant *t* by a network, which shows the relationship among the anatomical regions. This dynamic network representation will allow us to investigate the network properties of planning and execution subtasks.

Note also that the nodes, *V*, of the network are fixed to the active anatomic regions, and our goal is only to estimate the weights of the edges, *W*(*t*), of the brain network, *N*(*t*), for each time instance, *t* For this purpose we adopt the method suggested by our team in Kivilcim et al. (2018).

#### 3.2.2. Forming Local Meshes

It is well-known that the human brain operates with two contradicting principles, namely *locality* and *centrality*. Our suggested network model incorporates these two principals by defining a set of spatially local meshes then ensembling the local meshes to form the brain network. This representation not only avoids to define fully connected brain networks by omitting the connectivity among irrelevant brain regions but also reduces the computational complexity.

In order to define local meshes, for each window ***win***(***t***), we define the functional neighborhood matrix, Ω_*t*_, for each time instant *t*. The entries of Ω_*t*_ are binary, either 1 or 0, indicating if there is a connection between two regions or not. The size of the matrix is *M* × *M*, where *M* is the number of brain anatomical regions. The functional neighborhood matrix contains no self-connections, thus, Ω_*t*_(*i, i*) = 0∀*i* ∈ [1, *M*]. Recall that the brain regions are pruned by voxel selection. Thus, the regions which do not contain any voxels have no in/out connections, and the corresponding entries in Ω_*t*_ are all zero.

The connectivity of each region to the rest of the regions is determined by using Pearson correlation, as follows: first, for every region *i*, we measure the Pearson correlation between its BOLD response ***r***_***i,t***_ and the BOLD responses of all the other remaining regions as shown below:


(6) $$\begin{array}{l} cor\left(r_{i,t},r_{j,t}\right)=\frac{cov\left(r_{i,t},r_{j,t}\right)}{\sigma \left(r_{i,t}\right)\sigma \left(r_{j,t}\right)}, \end{array}$$

where ***r***_***i,t***_ is the BOLD response of region *i* across time window *win*(*t*), *cov*(***r***_***i,t***_, ***r***_***j,t***_) is the covariance between the corresponding BOLD responses of regions *i* and *j*. σ is the standard deviation of the BOLD response of a given region. Thus, the higher the Pearson correlation between two regions the closer they are to each other in the functional neighborhood system.

Then, we select *p* of the regions with the highest correlation scores with region *i*. Thus, a local mesh for each anatomic region *i* is formed by obtaining the neighborhood set η_*p*_[*i*], which contains the *p* closest brain regions to region *i*. The degree of neighborhood, *p*, is determined empirically as will be explained in the next section. Finally, we define the Ω_*t*_(*i, j*) as the connectivity between the regions *i* and *j*, using the constructed neighborhood sets as follows:


(7) $$\begin{array}{l} \Omega_{t}\left(i,j\right)=\{\begin{array}{cc} 1, & \text{if }j\in \eta_{p}\left[i\right]\\ 0, & \text{otherwise}. \end{array} \end{array}$$

Note that each anatomical region is connected to its *p* closest functional neighbors. This approach forms a star mesh around each anatomical region.

The ensemble of all of the local meshes creates a brain network at each time instance. Note, also, that Pearson correlation values are not used as the weights between two regions. They are just used to identify the nodes of each local mesh formed around an anatomical region. The estimated brain network becomes sparser as *p* gets smaller. When *p* is set to the number of anatomic regions, *M*, the network becomes fully connected. This approach of defining the connectivity matrix makes the network representation sparse for small *p*-values and constructs a network that is connected in functionally closest regions, satisfying the locality property of the human brain.

#### 3.2.3. Estimating the Edge Weights of the Brain Network

After having determined the edges of the brain graph using the functional neighborhood matrix Ω_*t*_, all that is left is to estimate the weights of these edges at each local mesh. At this point, we could use the Pearson correlation values as edge weights between two anatomic regions. However, Pearson values are restricted to measure the connectivity among the pairs only. A better approach is to consider the multiple relationships among an anatomic region and all of its neighbors in the local mesh. In order to estimate the edge weights in a mesh all at once, we represent the time series of each region *i* (***r***_***i,t***_) as a linear combination of its closest *p*-functional neighbors as shown in Equation (8):


(8) $$\begin{array}{l} {\hat{r}}_{i,t}=\underset{j\in \eta_{p}\left[i\right]}{\sum }w_{t,j,i}r_{j,t}+\epsilon_{i,t}. \end{array}$$

In Equation (8), ${\hat{r}}_{i,t}$ is the representative time series of of region *i* within the time window *win*(*t*), *w*_*t,j,i*_ is the estimated edge weight between node (region) *i* and node *j* at time instance *t*. η_*p*_[*i*] is the *p*-closest functional neighbors of region *i*.

Ertugrul et al. (2016) showed that representing the time series of an anatomic region as a linear combination of its closest neighbors provides better performance compared to using pairwise Pearson correlation in brain decoding. They estimated the arc-weights for each mesh formed around region *i* for each time window *win*(*t*) by minimizing the mean-squared error loss function using Ridge regression. In this approach, the mean-squared error loss function is minimized with respect to *w*_*t,j,i*_, for each mesh, independent of the other meshes, where the expectation is taken over the time-instances, in window *win*(*t*) as shown in Equation (9).


(9) $$\begin{array}{l} E\left[{\left(\epsilon_{i,t}\right)}^{2}\right]=E\left[{\left({\hat{r}}_{i,t}-\underset{j\in \eta_{p}\left[i\right]}{\sum }w_{t,j,i}r_{j,t}\right)}^{2}\right]+\lambda {\left\|w_{t,j,i}\right\|}^{2}, \end{array}$$

where λ is the L2 regularization parameter whose value is optimized using cross-validation. L2 regularization is used in order to improve the generalization of the constructed mesh networks. Note that the estimated arc-weights, *w*_*t,j,i*_ ≠ *w*_*t,i,j*_. Therefore, the ensemble of meshes yields a directed brain network.

In this study, we define an Artificial Neural Network to estimate the values of mesh arc-weights for all anatomical regions jointly in each time window, as proposed in Kivilcim et al. (2018). In this method, we estimate the mesh arc-weights matrix *W*(*t*) = {*w*_*t,j,i*_|*j,i* ∈ *V*} using a feed-forward neural network. The architecture of this network consists of an input layer and an output layer, both containing *M* nodes corresponding to each anatomic region. The edges of the feed-forward neural network are constructed using the neighborhood matrix Ω_*t*_. There is an edge between node *i* of the output layer and node *j* from the input layer, if Ω_*t*_(*i,j*) = 1.

The loss function of the suggested Artificial Neural Network is given in Equation (10), where *W* is the weight matrix of the entire neural network that corresponds to directed edge weights of the brain graph and *W*_*i*_ is the row of matrix *W* corresponding to region *i*:


(10) $$\begin{array}{l} Loss\left(Output_{i}\right)=E\left[{\left(\epsilon_{i,t}\right)}^{2}\right]+\lambda W_{i}^{T}W_{i}\\ \;=E\left[{\left(r_{i,t}-\underset{j\in \eta_{p}\left[i\right]}{\sum }w_{t,j,i}r_{j,t}\right)}^{2}\right]+\lambda W_{i}^{T}W_{i}. \end{array}$$

We train the aforementioned Artificial Neural Network in order to obtain the weights of the brain network at each time instance *t* that minimize the loss function by applying a gradient descent optimization method as shown in Equation (11),


(11) $$\begin{array}{l} w_{t,j,i}^{\left(\kappa \right)}=w_{t,j,i}^{\left(\kappa -1\right)}-\alpha_{learning}\frac{\partial E\left[{\left(\epsilon_{i,t}\right)}^{2}\right]}{\partial w_{t,j,i}}, \end{array}$$

where $w_{t,j,i}^{\left(\kappa \right)}$ is the weight of the edge from node *j* to node *i* at epoch (iteration) κ, α_*learning*_ is the learning rate. The number of epochs and learning rate used to train the network are optimized empirically using cross-validation.

Finally, the weights of the above artificial neural network, computed for each *win*(*t*), correspond to the edge weights of the dynamic brain network, *N*(*t*) = (*V, W*(*t*)), at each time instant *t*. Thus, we refer to the brain networks using their window indices in order to obtain a set of dynamic brain networks *T* = {*N*(1), *N*(2), …*N*(*tot*_*win*)}, where *N*(*t*) is the brain network for time window *win*(*t*) and *tot*_*win* is the total number of time windows.

### 3.3. Network Metrics for Analyzing Brain Networks

In this section, we introduce some measures which we will use to investigate the network properties of each phase of the complex problem solving task, namely, planning and execution, using the estimated dynamic brain functional networks. The connectivity patterns of anatomical regions are analyzed by the set of network measures given below. Two separate sets of measures are used, namely, measures of centrality and segregation. Since our estimated brain networks are directed, we distinguish the incoming and outgoing edges in the network while defining the measures.

Recall that the suggested brain network *N*(*t*) = (*V, W*(*t*)) consists of a set of nodes, *V*, each of which corresponding to one of the *M* anatomical regions. *W*(*t*) is the dynamic edge weight matrix with the entries, *w*_*i,j*_, representing the weight of the edge from node *i* to node *j*. For the sake of simplicity, we omit the time dependency parameter *t*, since we compute the network properties at each time instant.

#### 3.3.1. Measures of Centrality

Measures of centrality aim to identify brain regions that play a central role in the flow of information in the brain network or nodes that can be identified as hubs. It is commonly measured using node degree, node strength and node betweenness centrality, which are defined below.

##### 3.3.1.1. Node Degree

The degree of a node is the total number of its edges as shown in Equation (12), where *degree*_*i*_ is the degree of node *i*, *V* is the set of all nodes in the graph and *a*_*i,j*_ is the edge between node *i* and node *j*.


(12) $$\begin{array}{l} degree_{i}=\underset{j\in V}{\sum }a_{i,j}, \end{array}$$

where *a*_*i,j*_ takes value 0 if (*w*_*i,j*_ == 0) and takes value 1 otherwise.

In the case of a directed graph, we distinguish two different metrics: node in-degree $degree_{i}^{in}$ and node out-degree $degree_{i}^{out}$ metrics which are shown in Equations (13) and (14), respectively, where *a*_*j,i*_ = 1, if there is a directed edge from node *j* to node *i*.


(13) $$\begin{array}{l} degree_{i}^{in}=\underset{j\in V}{\sum }a_{j,i} \end{array}$$

(14) $$\begin{array}{l} degree_{i}^{out}=\underset{j\in V}{\sum }a_{i,j} \end{array}$$

Node degree is a measure of centrality of the given nodes, where it aims to quantify the hub brain regions interacting with a large number of brain regions. Thus, a node with high degree indicates its central role in the network.

##### 3.3.1.2. Node Strength

Node strength is the sum of the weights of edges connected to a given node (Equation 15), where *w*_*i,j*_ is the weight of the edge between node *i* and node *j*.


(15) $$\begin{array}{l} strength_{i}=\underset{j\in V}{\sum }w_{i,j} \end{array}$$

Similar to node degree, node strength, also, distinguishes two metrics in the case of directed graphs, namely, node in-strength $strength_{i}^{in}$ and out-strength $strength_{i}^{out}$ shown in Equations (16) and (17), respectively, where *w*_*j,i*_ is the weight of the edge from node *j* to node *i*.


(16) $$\begin{array}{l} strength_{i}^{in}=\underset{j\in V}{\sum }w_{j,i} \end{array}$$

(17) $$\begin{array}{l} strength_{i}^{out}=\underset{j\in V}{\sum }w_{i,j}. \end{array}$$

Node strength is a node centrality measure that is similar to node degree, which is used in the case of weighted graphs. Nodes with large strength values are tightly connected to other nodes in the network forming hub nodes.

##### 3.3.1.3. Node Betweenness Centrality

Betweenness centrality of node *i* is the fraction of the shortest paths in the network that pass through node *i* as shown in Equation (18)


(18) $$\begin{array}{l} betweenness_{i}=\frac{1}{\left(M-1\right)\left(M-2\right)}\underset{j,k\in V}{\sum }\frac{\rho_{j,k}^{i}}{\rho_{j,k}}, \end{array}$$

where ρ_*j,k*_ is the number of shortest paths between nodes *j* and *k*, $\rho_{j,k}^{i}$ is the number of shortest paths between nodes *j* and *k* that pass through node *i*, nodes *i*, *j* and *k* are distinct nodes.

Before measuring the betweenness centrality of a node, we need to change our perspective from connection weight matrix to connection length matrix since betweenness centrality is a distance-based metric. In connection weights matrix, larger weights imply higher correlation and shorter distance while it is the opposite in the case of length matrix. Connection length matrix is obtained by inverting the weights of the connection weight matrix. Then, the algorithm suggested in Brandes (2001) is employed in order to calculate the node betweenness centrality for each anatomical region.

Nodes with high betweenness centrality are expected to participate in many of the shortest paths of the networks. Thus, taking a crucial role in the information flow of the network.

#### 3.3.2. Measures of Segregation

Measures of segregation aim to quantify the existence of subgroups within brain networks, where the nodes are densely interconnected. These subgroups are commonly referred to as clusters or modules. The existence of such clusters in functional brain networks is a sign of interdependence among the nodes forming the cluster. Measures of segregation include clustering coefficient, transitivity and local efficiency. While global efficiency is a measure of functional integration representing how easy it is for information to flow in the network.

##### 3.3.2.1. Clustering Coefficient

The clustering coefficient of a node *i* is the fraction of triangles around node *i* which is calculated by Equation (19) as proposed in Fagiolo (2007). It is defined as the fraction of the neighbors of node *i* that are also neighbors of each other.


(19) $$\begin{array}{l} C_{i}=\frac{\chi_{i}}{\left[\left(d_{i}^{out}+d_{i}^{in}\right)\left(d_{i}^{out}+d_{i}^{in}-1\right)-2\sum_{j\in V}a_{i,j}a_{j,i}\right]}. \end{array}$$

where $d_{i}^{in}$ is the in-degree of node *i* and $d_{i}^{out}$ is the out-degree of node *i*. χ_*i*_ is the weighted geometric mean of triangles around node *i* that is calculated by Equation (20). Recall that *a*_*j,i*_ = 1, if there is a directed edge from node *j* to node *i* and *a*_*j,i*_ = 0, otherwise.


(20) $$\begin{array}{l} \chi_{i}=\frac{1}{2}\underset{j,h\in V}{\sum }{\left(w_{i,j}w_{i,h}w_{j,h}\right)}^{1/3}. \end{array}$$

The clustering coefficient of a node is the fraction of triangles around the node. It is defined as the fraction of the neighbors of the node that are also the neighbors of each other.

##### 3.3.2.2. Transitivity

Transitivity of a node is similar to its clustering coefficient. However, transitivity is normalized over all nodes, while cluster coefficient for each node is normalized independently, which makes clustering coefficient biased toward nodes with low degree. Transitivity can be expressed as the ratio of triangles to triplets in the network. It is calculated by Equation (21), as suggested in Fagiolo (2007):


(21) $$\begin{array}{l} T_{i}=\frac{\chi_{i}}{\sum_{j\in V}\left[\left(d_{j}^{out}+d_{j}^{in}\right)\left(d_{j}^{out}+d_{j}^{in}-1\right)-2\sum_{h\in V}a_{j,h}a_{h,j}\right]} \end{array}$$

where $d_{j}^{in}$ is the in-degree of node *j* and $d_{j}^{out}$ is the out-degree of node *j*. χ_*i*_ is the weighted geometric mean of triangles around node *i* that is calculated by Equation (20). Note that *a*_*h,j*_*a*_*j,h*_ = 1, if there exits an edge in both directions.

##### 3.3.2.3. Global and Local Efficiency

The global efficiency of a brain network is a measure of its functional integration. It measures the degree of communication among the anatomical regions. Thus, it is closely related to the small-world property of a network. Formally speaking, global efficiency is defined as the average of the inverse shortest path lengths between all pairs of nodes in the brain network. Equation (22) shows how to calculate the global efficiency of a brain network, where $\varrho_{i,j}^{w}$ is the weighted shortest path length between two distinct nodes *i* and *j* (Rubinov and Sporns, 2010).


(22) $$\begin{array}{l} E_{global}=\frac{1}{M}\underset{i\in V}{\sum }\frac{\sum_{j\in V}{\left(\varrho_{i,j}^{w}\right)}^{-1}}{M-1} \end{array}$$

On the other hand, the local efficiency of a network is defined as the global efficiency calculated over the neighborhood of a single node. The local efficiency is, thus, a measure of segregation rather than functional integration as it is closely related to clustering coefficient. While global efficiency is calculated for the entire network, local efficiency is calculated for each node in the network (Rubinov and Sporns, 2010).

## 4. Experiments and Results

In this section, we explore the validity of the suggested dynamic brain network model and study the network properties of complex problem solving task on TOL dataset. First, we analyze the effect of the preprocessing step on the brain decoding performance of planning and execution phases of complex problem solving. Then, we investigate the validity of the dynamic functional brain network model proposed in this study. Finally, we analyze the network properties of the constructed functional brain networks for planning and execution subtasks.

### 4.1. Voxel Selection

**Figure 4:**
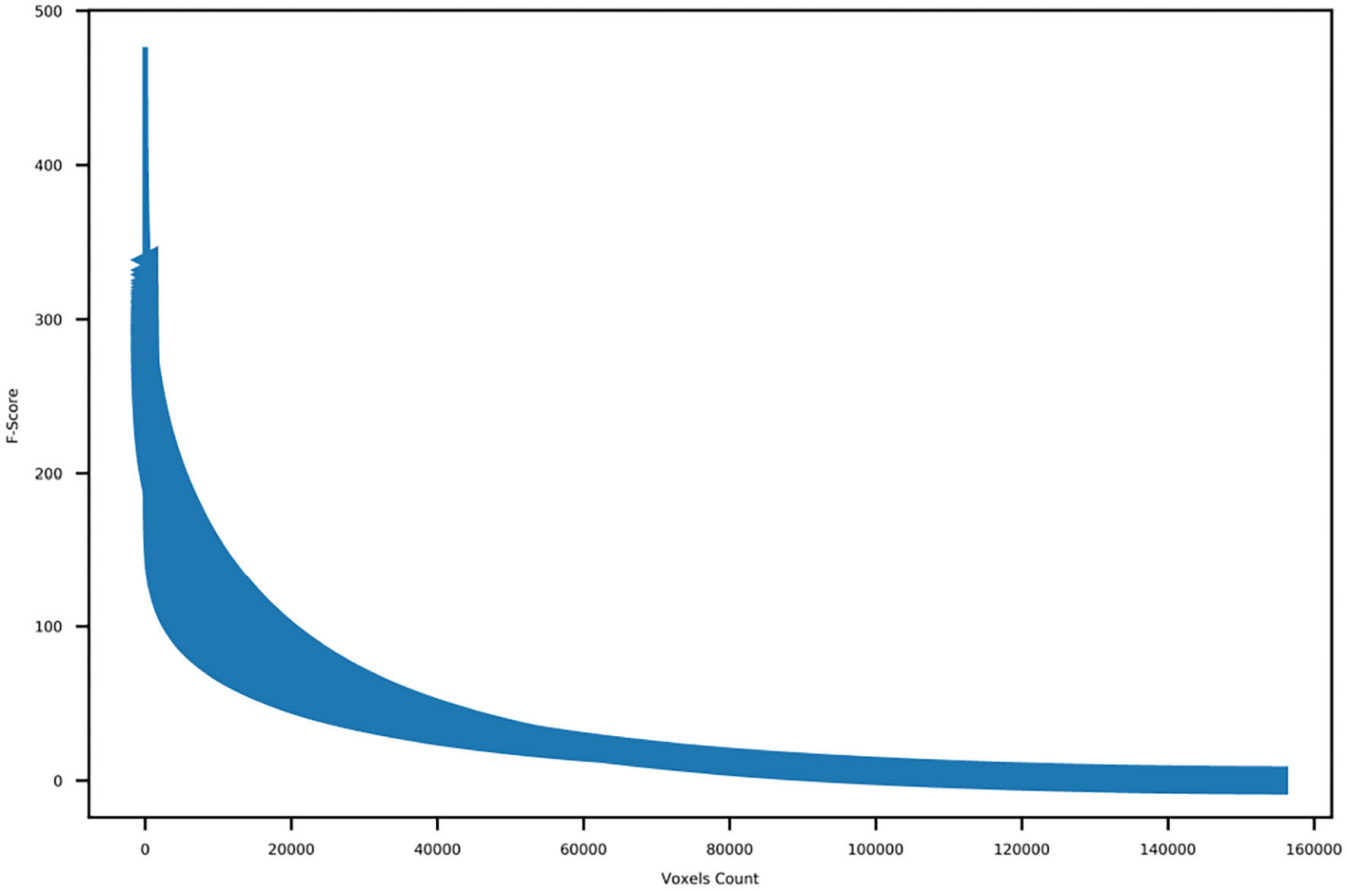
Ordered *f*-scores of voxels for all subjects.

First, we discarded all of the voxels located in the cerebellum anatomical regions. Then, we calculated the *f*-score for each one of the remaining voxels and order the obtained *f*-scores of the voxels. Following that, we plotted the ordered *f*-scores of the voxels in order to determine the appropriate number of voxels to retain. Figure 4 shows the ordered *f*-scores of the voxels averaged across all subjects. It can be observed from this figure that a relatively small number of voxels is crucial for discriminating the subtasks of problem solving while the remaining voxels do not have significant information concerning the subtasks of problem solving. Based on the *f*-score distribution shown in Figure 4, we kept the 10,000 voxels with the highest *f*-scores observing the elbow point, whereas we discarded the remaining ones.

**Figure 5:**
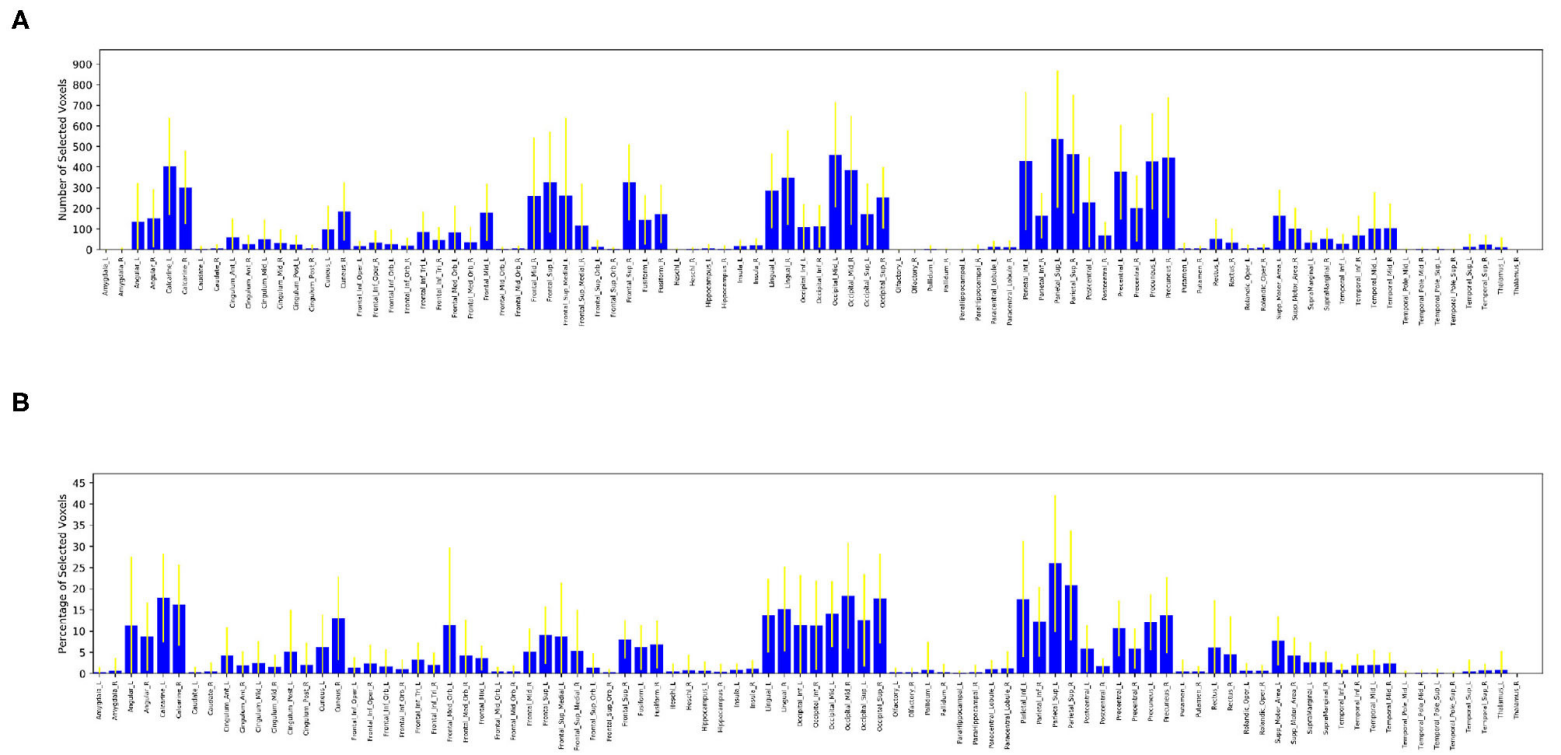
Distribution of selected voxels across anatomical regions, measured by number of selected voxels (top) and percentage of selected voxels (bottom) from each anatomical region. A large value in both figures is an indication of relatively high activity in a particular anatomic region. **(A)** Average number of voxels selected from each anatomical region across all subjects. **(B)** Average percentage of voxels selected from each anatomical region across all subjects.

After selecting 10,000 voxels with the highest *f*-scores of each run, we calculated the number of selected voxels contained in each one of the 90 anatomical regions. We also calculated the percentage of selected voxels to the total number of voxels located in each anatomical region. These values of selected voxels can be considered as measures of participation of an anatomic region into the complex problem solving task. The Figure 5A shows the average number of voxels contributed by each region across all subjects with its corresponding standard deviation, Figure 5B shows the average percentage of voxels contributed by each region across all subjects with its corresponding standard deviation.

It is clear from these figures that a large number of regions contribute little to no voxels, such as the amygdala, caudate, heschl gyrus, hippocampus, pallidum, putamen, temporal pole, superior temporal cortex, thalamus and parahippocampus. A small number of regions contribute a significantly large number of voxels (over 300 voxels each) during complex problem solving, such as occipital, precentral, precuneus and parietal regions.

Furthermore, Figure 5B ensures that there is no bias against tiny anatomical regions with small number of voxels by normalizing the number of voxels selected from each region by its total number of voxels. Figure 5B clearly shows that in the *left prefrontal and inferior occipital* regions a significant percentage of voxels are active during complex problem solving. Both figures also show high standard deviations across subjects, which indicates high inter-subject variability.

### 4.2. Interpolation

**Figure 6:**
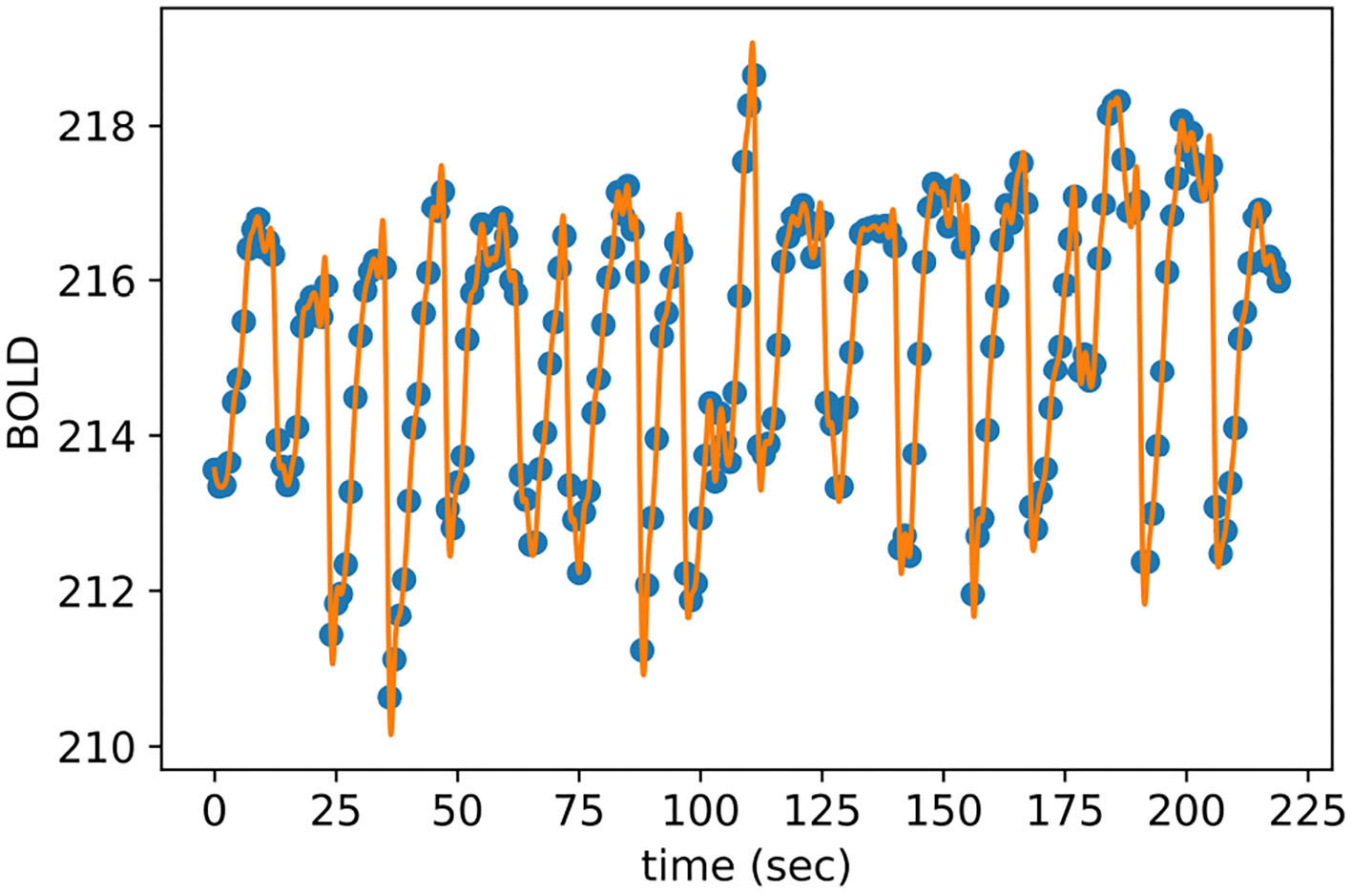
Interpolated BOLD response of a randomly selected anatomic region.

After selecting the most discriminative voxels and averaging their BOLD responses with respect to their corresponding brain anatomical regions, we employed temporal interpolation to each representative time series to increase the temporal resolution of the TOL dataset. As a result, the total number of obtained brain volumes is equal to *n* + *z* * (*n* − 1) where *n* is the number of measured brain volumes of a given puzzle and *z* is the number of estimated brain volumes plugged between each pair of measured brain volumes. The optimal value of *z* is equal to 8, which is determined empirically using cross-validation to maximize the brain decoding performance. Figure 6 shows the interpolated BOLD response of a randomly selected anatomical region from the given subjects, where the blue dots represent the measured BOLD response of the region and the orange dashes are the interpolated values. It is clear from Figure 6 that the interpolated points using cubic spline function do not introduce sharp edges, nor do they smooth out the spikes between measured brain volumes.

**Figure 7:**
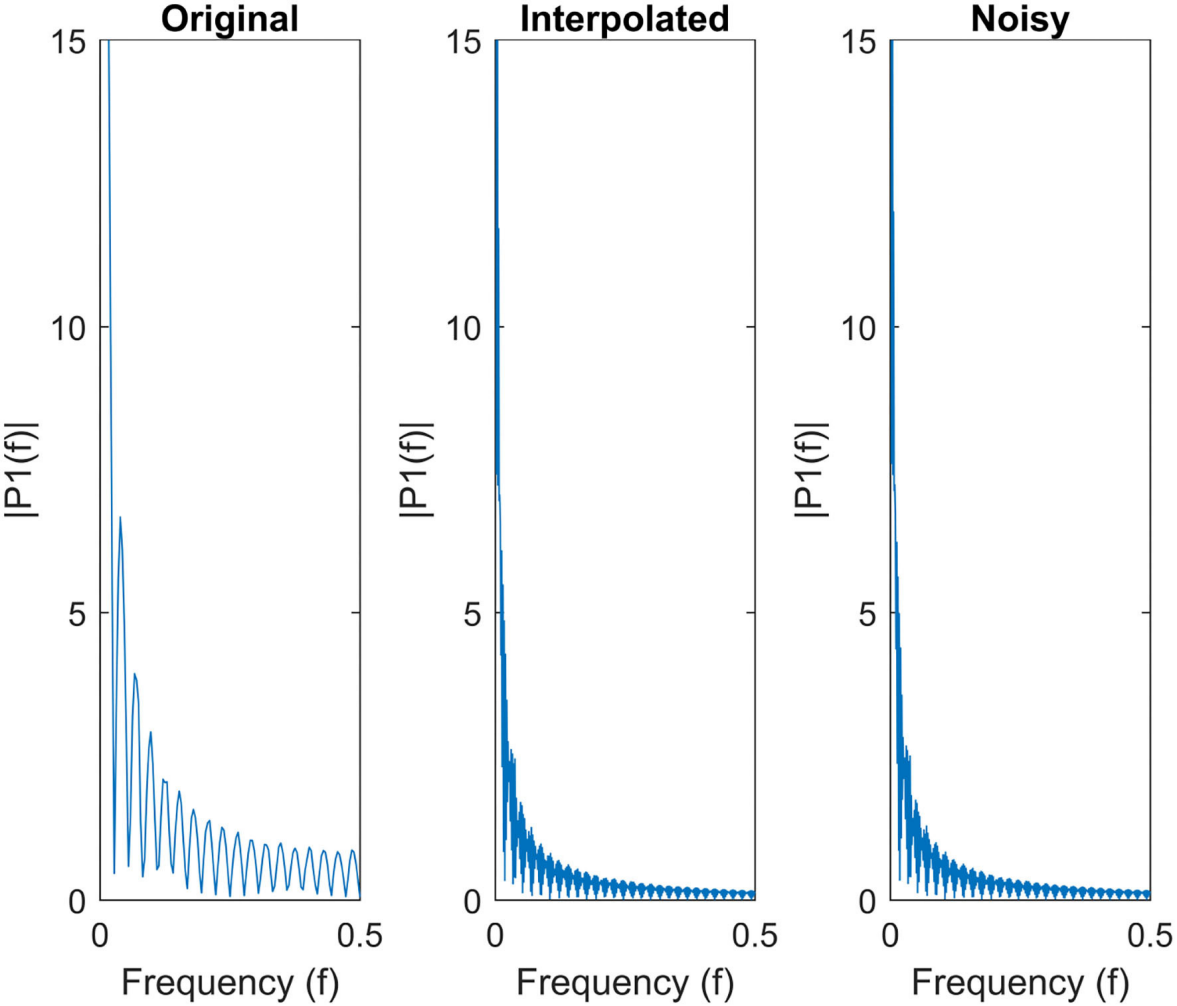
Single-Sided amplitude spectrum of a time series of an randomly selected anatomic region.

Furthermore, Figure 7 shows the single-sided amplitude spectrum of a randomly selected anatomical region from a given subject before interpolation, after interpolation and finally, after adding Gaussian noise. The figure clearly demonstrates that both interpolation and injecting Gaussian noise preserve the envelope of the signal in the frequency domain.

### 4.3. Gaussian Noise

In order to control the signal-to-noise ratio (SNR), we used cross-validation to choose the optimal pair of values for α_*noise*_ and β_*noise*_, the ratios of mean and standard deviation of the added noise, respectively. As a result, the optimal values obtained are α_*noise*_ = 0.025 and β_*noise*_ = 0.075 from the following set of values α_*noise*_, β_*noise*_ ∈ [0.025, 0.05, 0.075, 0.1].

### 4.4. Brain Decoding With Preprocessed fMRI Data

We use brain decoding in order to investigate the validity of our proposed preprocessing steps on the TOL dataset. We aim to distinguish the two phases of complex problem solving, namely: planning and execution. At first, we used ANOVA to select the 10,000 voxels with the highest *f*-scores. Then we averaged the selected voxels into their corresponding anatomical regions defined by Tzourio-Mazoyer et al. (2002). Following that, we employed temporal interpolation to increase the temporal resolution of each puzzle by estimating *z* = 8 brain volumes between each pair of measured brain volumes. Finally, we added Gaussian noise in order to regularize the BOLD responses of each region to improve the generalization performance of the classifiers. We used *k*-fold Cross validation for each subject in all of the experiments introduced in this section, with *k* = 8. After we obtained the results, we averaged them across the different folds, then we calculated the average and standard deviation across all subjects. We used both supervised and unsupervised brain decoding methods. A linear support-vector machine (SVM) (Fan et al., 2008) was used for supervised brain decoding while *k*-means clustering was used for unsupervised brain decoding. The input to the decoders is formed by concatenating the values of representative time series computed per each time instant, across the anatomic regions. Considering the fact that there is a total of 90 anatomic regions, the dimension of the input vectors is 90. If there are no selected voxels in an anatomic region after the voxel selection process, the corresponding entry of the input vector becomes 0.

Table 2 shows the effect of our preprocessing pipeline on the brain decoding of complex problem solving subtasks. The first row shows the performances of brain decoding on the raw dataset without any preprocessing, simply averaging all of the voxels into their corresponding anatomical regions. While the second row shows the results of applying voxel selection then averaging the selected voxels into their anatomical regions. The third row shows the results of brain decoding after applying temporal interpolation, while the forth row shows the results after injecting the data with Gaussian noise.

**Table 2:** Decodinge performances of preprocessing pipeline after each step.

**Preprocessing**	**SVM**	**k-Means**
Raw data	0.60 ± 0.11	0.63 ± 0.09
Voxel selection	0.74 ± 0.12	**0.85** ± 0.06
Interpolation	0.81 ± 0.08	0.84 ± 0.06
Noise addition	**0.82** ± 0.08	**0.85** ± 0.06

*Bold is used to indicate the best values obtained across a given column (i.e. the highest accuracy obtained)*.

From the results of the preprocessing experiments, it is observed that voxel selection improves the brain decoding performance for both supervised and unsupervised methods from 60 to 74% and from 63 to 85%, respectively. This can be attributed to the fact that voxel selection retains only the most discriminative voxels and trashes the remaining non informative ones. In addition, voxel selection manages to sparsify the representation of the data since some brain regions contribute no voxels at all; thus have no contribution to brain decoding.

The table also shows that temporal interpolation further improves the supervised brain decoding performance from 74 to 81%; this significant increase is due to increasing the number of brain volumes, thus, increasing the number of training samples for the SVM classifier. However, temporal interpolation slightly reduces the performance of unsupervised methods from 85 to 84%. This result can be partially attributed to the fact that the additional brain volumes smooth the mixture distribution, thus reducing the distinction between the two phases of problem solving, planning and execution. This is due to the method used to label the estimated brain volumes, where each estimated brain volume is given the labels of its closest neighboring measured brain volume.

Finally, the addition of Gaussian noise slightly boosts the performance of both supervised and unsupervised methods from 81 to 82% and from 84 to 85%, respectively. The table also shows high standard deviation across subjects, which is consistent with voxel selection plots, revealing high inter-subject variability.

### 4.5. Brain Decoding With Dynamic Brain Networks

In this section, we compare our model for building dynamic functional brain networks with some of the popular network methods proposed in the literature in terms of their brain decoding performance. Brain decoding performance can be considered as a measure of validity of the proposed brain networks. High decoding performance indicates that the constructed brain network has a good representation power of the underlying cognitive subtasks, namely planning and execution.

For this purpose, we built the dynamic brain networks, as explained in the previous sections, after having successfully applied the preprocessing pipeline. It is important to remark that each time instance has either a planning label or an execution label. While constructing the brain networks, we define a feature vector for each time instance by using the interpolated time instances (4 extra instances at each side of a measured instance). Thus, for each measured time sample, we form 4+4+1= 9 brain volumes to estimate the brain network weights. These weights represent a network among 90 anatomic regions for each measured time instance.

The optimal values for learning rate α_*learning*_ and the number of epochs were chosen empirically using cross-validation, obtaining the following values, respectively 1 * 10^−8^ and 10. As for *p*, the number of neighbors used to represent each anatomical region; we chose *p* equal to the total number of regions, which is 90. In this way, a fully-connected brain network is obtained at each time window. However, the total number of nodes is less than 90, given that some regions have flat BOLD responses; therefore, they were pruned along with all their edges from the brain network.

We also constructed brain networks using Pearson correlation and ridge regression as proposed in Richiardi et al. (2011), Onal et al. (2015), Ertugrul et al. (2016), and Onal et al. (2017), respectively, in order to compare the performance of our methods with other works in the literature. In the case of Pearson correlation, the functional brain networks were constructed using Pearson correlation scores between each pair of brain regions (Richiardi et al., 2011; Ertugrul et al., 2016). As for the case of ridge regression, the mesh arc-weight descriptors were estimated using ridge regression in order to represent each region as a linear combination of its neighbors (Onal et al., 2015, 2017).

Since our goal is to represent the fMRI data by an informative dynamic network structure, we used generic classification/clustering methods with relatively small learning capacity in order to highlight the representative power of the constructed brain networks. For this reason, we used simple classifiers/clustering methods, such as SVM and K-means. It would be possible to improve the brain decoding performances by using methods with higher learning capacity, such as Multi-Layer Perceptrons. In this case, the dynamic network representation of the Artificial Neural Networks is expected to obtain much better performances compared to the ones reported in the paper. However, the reported performances are sufficient to show that the decoding performance of the dynamic network, which is 90 x 90 = 8,100 edges of the brain network, is compatible with the fMRI data, although it is much more compact and more informative than the raw data (185,000 voxels per brain volume).

The main advantage of representing the fMRI data by dynamic brain networks is that they are neuroscientifically interpretable and much more comprehensive compared to the voxel based representations. The constructed dynamic brain networks allow us to investigate a large variety of network properties in order to identify regions of interest, such as hubs and subgroups of densely connected brain regions with the aim of deriving neuro-scientifically valid insights into the planning and execution phases of the complex problem solving task.

Table 3 shows the brain decoding results of the aforementioned brain network construction methods compared against the results of multi-voxel pattern analysis (MVPA), which feeds the preprocessed BOLD response representing a time instant of all brain regions into a classifier. The first row shows the brain decoding results of preprocessed fMRI data, where there is no network representation at all. This data is obtained by applying voxel selection, interpolation then noise addition to the raw fMRI data. The first row of Table 3 is the same as the last row of Table 2.

**Table 3:** Braine decoding performances of proposed dynamic brain network model compared to the state of the art models, namely, pearson correlation and ridge regression.

**Input to algorithm**	**SVM**	**k-Means**
Preprocessed fMRI Data	**0.82** ± 0.08	0.85 ± 0.06
Pearson correlation	0.58 ± 0.05	0.57 ± 0.04
Ridge regression	0.56 ± 0.05	0.55 ± 0.02
Dynamic brain networks	**0.82** ± 0.10	**0.87** ± 0.06

*Bold is used to indicate the best values obtained across a given column (i.e. the highest accuracy obtained)*.

The second and third rows show the brain decoding performances of the networks extracted using Pearson correlation and Ridge regression methods, respectively. The Pearson Correlation data is generated using the preprocessed fMRI data, where Pearson correlation is used to generate the brain networks. The weights of the edges in the constructed brain networks are the Pearson correlation scores between the preprocessed BOLD responses of the corresponding anatomic regions (as shown in Equation 6). The Ridge Regression data is generated using the preprocessed fMRI data, where Ridge Regression is used to estimate the weights of the edges in the constructed brain networks. The weights of the edges in the constructed brain networks are estimated using Ridge regression by minimizing the cost function of Equation (9).

The last row shows the brain decoding performances of our proposed Dynamic Brain Network model.

The edge weights of the Dynamic Brain Network is computed by training an Artificial Neural Network with the preprocessed fMRI data. The nodes in the dynamic brain networks represent the brain anatomic region. The edge links of the brain networks are determined by using Equation (7). The edge weights are estimated using Artificial Neural Network as shown in Equations (10) and (11).

The inputs to SVM and K-Means in the case of Pearson Correlation, Ridge Regression and Artificial Neural Networks are the estimated weights of the brain networks. A feature vector of edge weights, with 90 x 90 = 8,100 dimension, is defined at each recorded time instance of fMRI data, as a single training/testing sample. Each feature vector has its corresponding class label, as Planning or Execution.

Table 3 clearly shows that both Pearson correlation and Ridge regression fail to construct valid brain networks that are good representatives of the underlying cognitive tasks. However, our model managed to get brain decoding results similar or slightly better than those obtained from MVPA both in the cases of supervised and unsupervised methods. This can be attributed to the challenging nature of the TOL dataset; Pearson correlation does not manage to capture the interdependencies between the anatomical regions over short time windows. While ridge regression fails to correctly estimate the mesh arc-weights as it estimates the arc-weights for each region independently of the other ones. Our proposed model, with a relatively small number of epochs, manages to obtain mesh arc-weight values that capture the activation patterns of anatomical regions and their relationships.

It is important to note that, it is possible to obtain higher brain decoding accuracy using voxel-level MVPA rather than anatomic-region-level MVPA, and by normalizing the BOLD response of individual voxels to having 0 mean and 1 standard deviation. However, we do not employ either of them in our analysis for the following reasons. Firstly, any analysis at the voxel-level comes with a very high computational cost, especially when attempting to build functional brain networks. Also, the analysis at voxel level does not allow us to perform the study roles and contributions of brain anatomic region level to the complex problem solving task, which is the main goal of this paper. Secondly, normalizing the BOLD responses of individual voxels prevents us from constructing informative, functional brain networks as this normalization distorts the information of relative activation patterns between the voxels and the brain anatomic region, which is essential in the process of building functional brain networks.

## 5. Brain Network Properties

In this section, we aim to analyze the network properties of the constructed functional brain networks. We investigate the network properties for each anatomical brain region during both planning and execution subtasks in order to understand which regions are most active and which regions work together during each one of the two subtasks of complex problem solving.

Given that the constructed brain functional networks are both weighted, directed, fully-connected and contain both negative and positive weights, we preprocessed the networks before measuring their network properties. Firstly, we got rid of all the negative weights by shifting all the mesh arc-weights values by a positive quantity equal to the absolute value of the largest negative arc-weight. We then normalized the mesh arc-weights to ensure that all of the weights are within the range of [0, 1]. Finally, we measured the network properties on the pruned brain graph, where the brain regions (nodes) contributing no voxels (have a flat BOLD response) and all of their corresponding arc-weights (edges) were deleted from the brain graph. Thus, the networks contained less than 90 regions with their corresponding edges. We used the brain connectivity toolbox to calculate the investigated network properties (Rubinov and Sporns, 2010).

In order to measure for centrality, the number of neighbors for each anatomical region (P) was chosen to be equal to 89, which is equal to the total number of neighbors for any given node as the total number of brain anatomical regions defined by the AAL atlas (Tzourio-Mazoyer et al., 2002) after deleting the regions residing in the cerebellum equals 90. In addition, since we pruned the nodes that correspond to regions from which no voxels were selected, our constructed brain networks were weighted directed fully-connected networks. Therefore, the in-degree, out-degree and total degree of all nodes in the graph were equal to the total number of anatomical regions retained after voxel selection.

Therefore, we used node strength and node betweenness centrality to identify nodes with high centrality, which are potential hubs in the brain networks controlling the flow of information in the network. In our proposed model, the node in-strength of node *i* is the sum of the mesh arc-weight values, which is estimated using our proposed neural network method in order to minimize the reconstruction error of the BOLD response of anatomical region *i* using its neighbors. Thus, node in-strength is not used as part of our network properties analyses; we rather used node out-strength to measure the centrality of all anatomicalregions.

As for measures of segregation, quantifying the existence of subgroups within brain networks is based on densely interconnected nodes. These subgroups are commonly referred to as clusters or modules. The existence of such clusters in functional brain networks is a sign of interdependence among the nodes forming the cluster. Therefore, clustering coefficient, transitivity and local efficiency were measured in order to identify potential clusters with dense interconnections in the brain networks.

### 5.1. Planning and Execution Brain Networks

In this section, we discuss the network properties of the planning and execution networks. For each aforementioned network metric, we ranked the brain regions in descending order according to their score on that network measure for all subjects across all runs. Then, we retained the 10 anatomical regions with the highest scores. Following that, we measured the frequency of occurrence of each brain region among the top 10 regions across all runs in order to identify the shared regions and patterns across all subjects for both planning and execution subtasks. The results of the analysis are shown in tables: Table 4 shows the brain regions that have high scores for the reported network properties during planning subtask, and Table 5 shows the brain regions that have high scores during execution subtask. There are a number of processes taking place during planning and execution. Plan generation involves a series of recursive events including 1) problem encoding; 2) decision-making in order to decide which ball to move and where to move it; 3) mental imagery to imagine the ball moving, and 4) working memory to maintain the intermediate steps as well as the move number. During plan execution, there is 1) retrieval of the steps from memory; 2) confirming the correct steps are being performed, and 3) the motor execution of those steps. As the results demonstrate, the networks for planning and execution are overlapping. These results are similar to the activation results reported in Newman et al. (2009) in that the regions that were found to be active during the task are also regions that are most prominently found with the highest network measures. These regions include the right and left middle frontal gyrus, anterior cingulate cortex, precentral cortex, and superior parietal cortex.

**Table 4:** Planning: Anatomical regions with the highest network measures across subjects, regions are painted if they overlap with execution.

**Transitivity**	**Local efficiency**	**Clustering Coefficient**	**Betweenness**	**Out-strength**
Angular	Calcarine	Calcarine	Cueneus R	Cueneus R
Calcarine	Cuneus	Cuneus	Frontal Sup L	Frontal Sup L
Cingulum Ant	Frontal Mid R	Frontal Mid R	Fusiform R	Fusiform R
Cingulum Mid	Frontal Sup	Frontal Sup	Paracentral Lobule L	Paracentral Lobule L
Cuneus	Fusiform	Fusiform	Parietal Sup R	Supp Motor Area R
Frontal Inf Oper L	Occipital Inf R	Occipital Inf R	Precuneus L	Temporal Inf R
	Precentral	Parietal Sup R	Supp Motor Area R	Temporal Mid R
	Supp Motor Area R	Precentral	Temporal Inf R	
	Temporal Inf R	Supp Motor Area R	Temporal Mid R	
		Temporal Inf R		

**Table 5:** Execution: Anatomical regions with the highest network measures across subjects, regions are painted if they overlap with planning.

**Transitivity**	**Local efficiency**	**Clustering coefficient**	**Betweenness**	**Out-strength**
Angular	Calcarine	Calcarine	Cueneus R	Cueneus R
Calcarine	Cuneus	Cuneus	Frontal Sup L	Frontal Sup L
Cingulum Ant	Frontal Mid R	Frontal Mid R	Fusiform R	Fusiform R
Cingulum Mid	Frontal Sup	Frontal Sup	Paracentral Lobule L	Paracentral Lobule L
Cuneus	Fusiform	Fusiform	Parietal Sup R	Supp Motor Area R
Frontal Inf Oper L	Occipital Inf R	Occipital Inf R	Precuneus L	Temporal Inf R
	Precentral	Parietal Sup R	Supp Motor Area R	Temporal Mid R
	Supp Motor Area R	Precentral	Temporal Inf R	
	Temporal Inf R	Supp Motor Area R	Temporal Mid R	
		Temporal Inf R		

**Figure 8:**
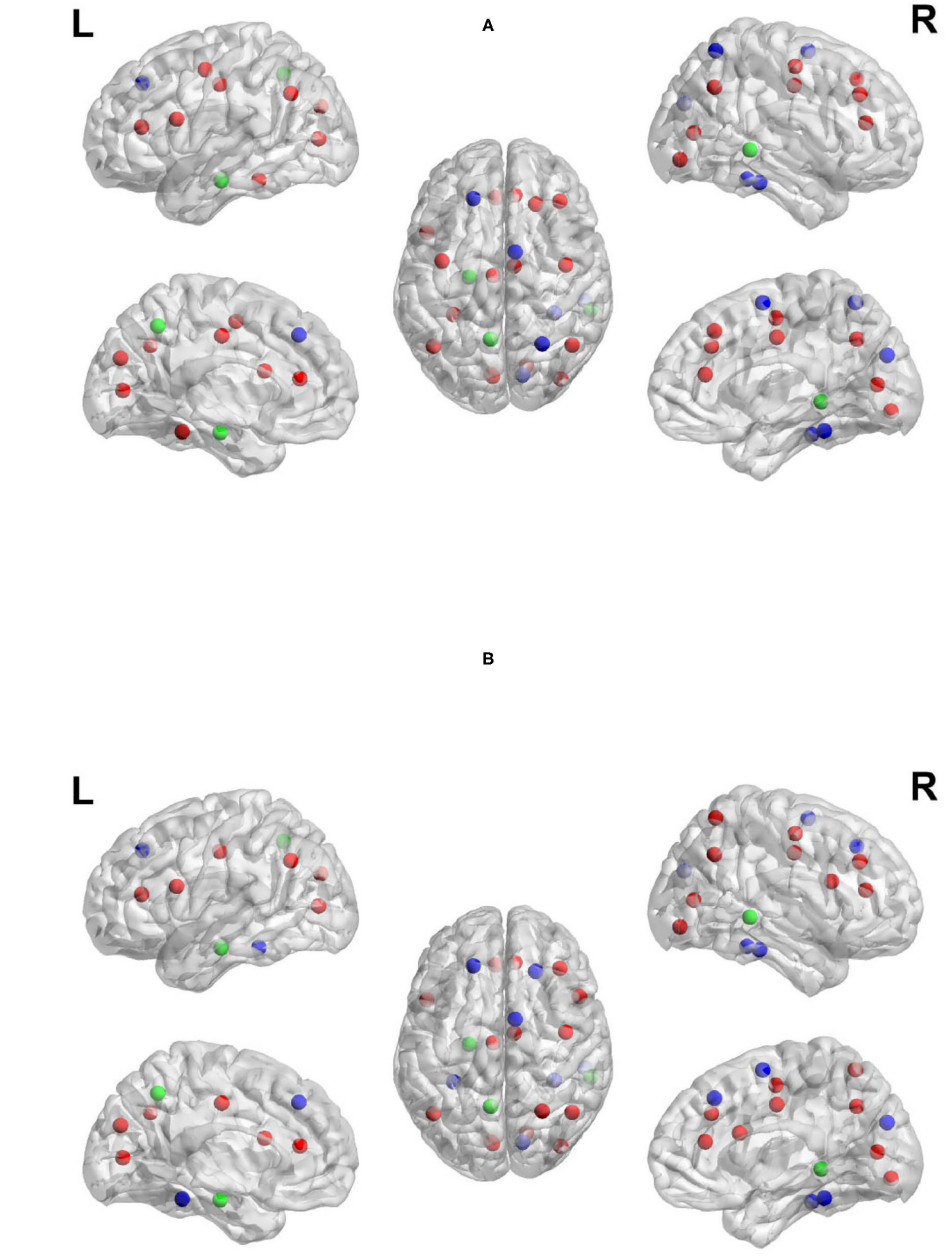
Regions with the highest network measures for planning brain network **(A)** and execution brain network **(B)**.

Previous work has suggested that the regions found in the current study to show high network measures are directly related to the sub-tasks associated with TOL performance. For example, both the left and right prefrontal cortex have been found to be involved in the TOL task, with the two regions performing distinguishable functions. The right prefrontal cortex is involved in constructing the plan for solving the TOL problem while the left prefrontal cortex is involved in supervising the execution of that plan (Newman et al., 2003, 2009). The anterior cingulate has been linked to error detection and is particularly involved in the TOL when the number of moves is higher, or the problem difficulty is manipulated. The right superior parietal cortex and precentral cortex have been linked to visuo-spatial attention necessary for planning (Newman et al., 2003), and the left parietal cortex has been linked to visuo-spatial working memory processing (Newman et al., 2003). The overlap between the regions with the highest network measures and those that have been linked to the task is an important feature and is not due to the voxel selection process. Many regions that passed threshold were not in the top ranked list of network measures. For example, the basal ganglia, including the caudate has been found in previous studies to be involved in TOL performance (Dagher et al., 1999; Rowe et al., 2001; Beauchamp et al., 2003; Van den Heuvel et al., 2003; Newman et al., 2009); however, the region appears to not be an important network hub. Figures 8A,B visualize the reported brain regions in Tables 4, 5, respectively, using Brain Net Viewer (Xia et al., 2013). In Figures 8A,B, the color of the node (brain region) implies the following: red indicates that the region has high transitivity, clustering coefficient or local efficiency. Green indicates that the node has high node centrality measured by node out-strength and node betweenness. As for blue, it shows the nodes that have high node centrality and is part of a subgroup of densely interconnected regions.

### 5.2. Differences Between Planning and Execution Networks

In this section, we explore the network differences between planning and execution by calculating the difference between the network property scores for planning and execution for each run. To achieve that, we took the difference between the network property scores for brain anatomical regions during planning and the network property scores for brain anatomical regions during execution for each run. Then, we counted the frequency of times a given anatomical region is more active during planning than execution and vice-versa in order to identify consistent patterns of the disagreements between planning brain networks and execution brain networks across all subjects. Results showed, generally, that the network measures were higher for planning than execution. This, too, mirrors the findings from Newman et al. (2009) in which planning resulted in greater activation than execution.

**Figure 9:**
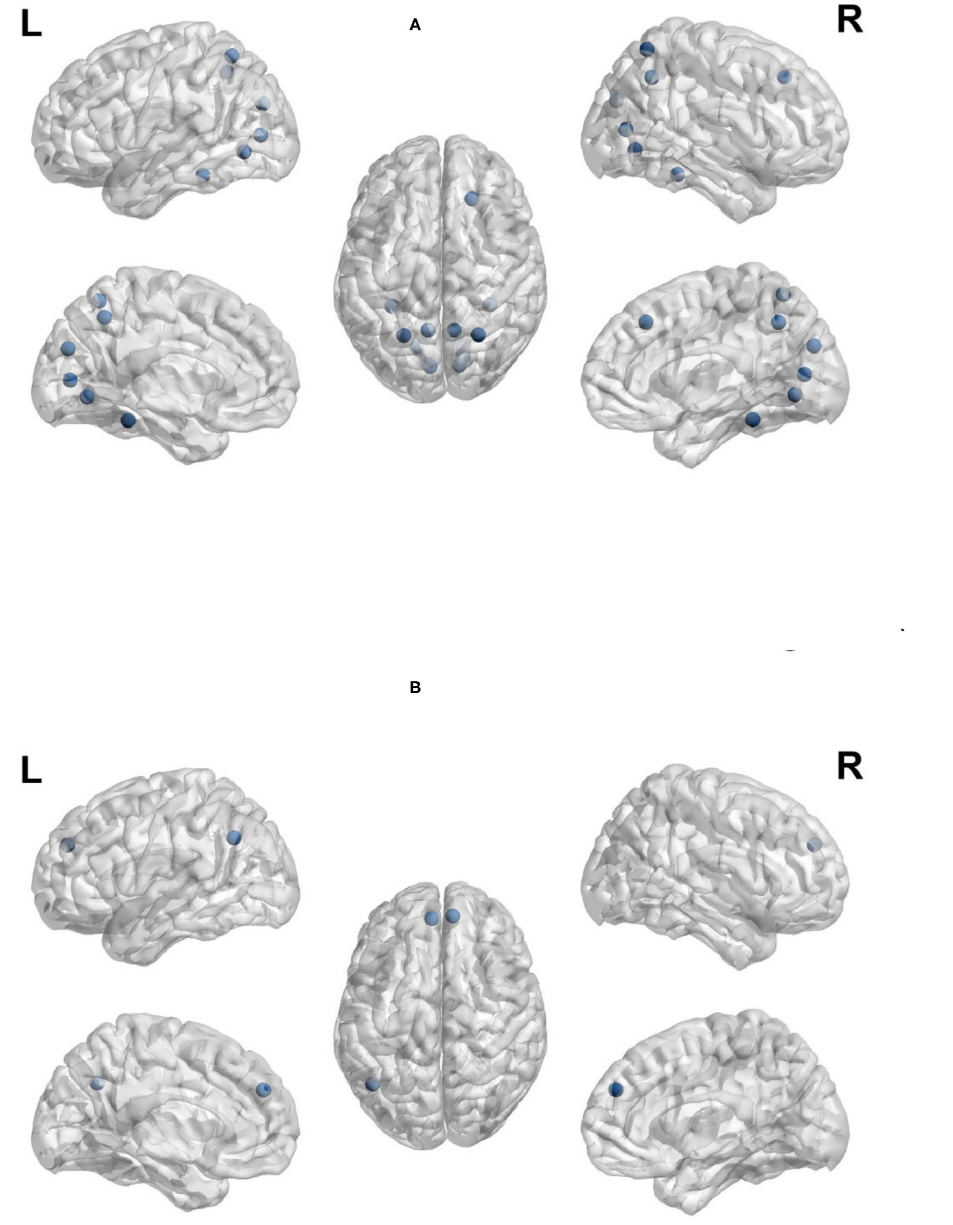
Anatomical regions with higher node out-strength during planning (Top) and during execution (Bottom). **(A)** Anatomical regions with higher node out-strength during planning. **(B)** Anatomical regions with higher node out-strength during execution.

**Figure 10:**
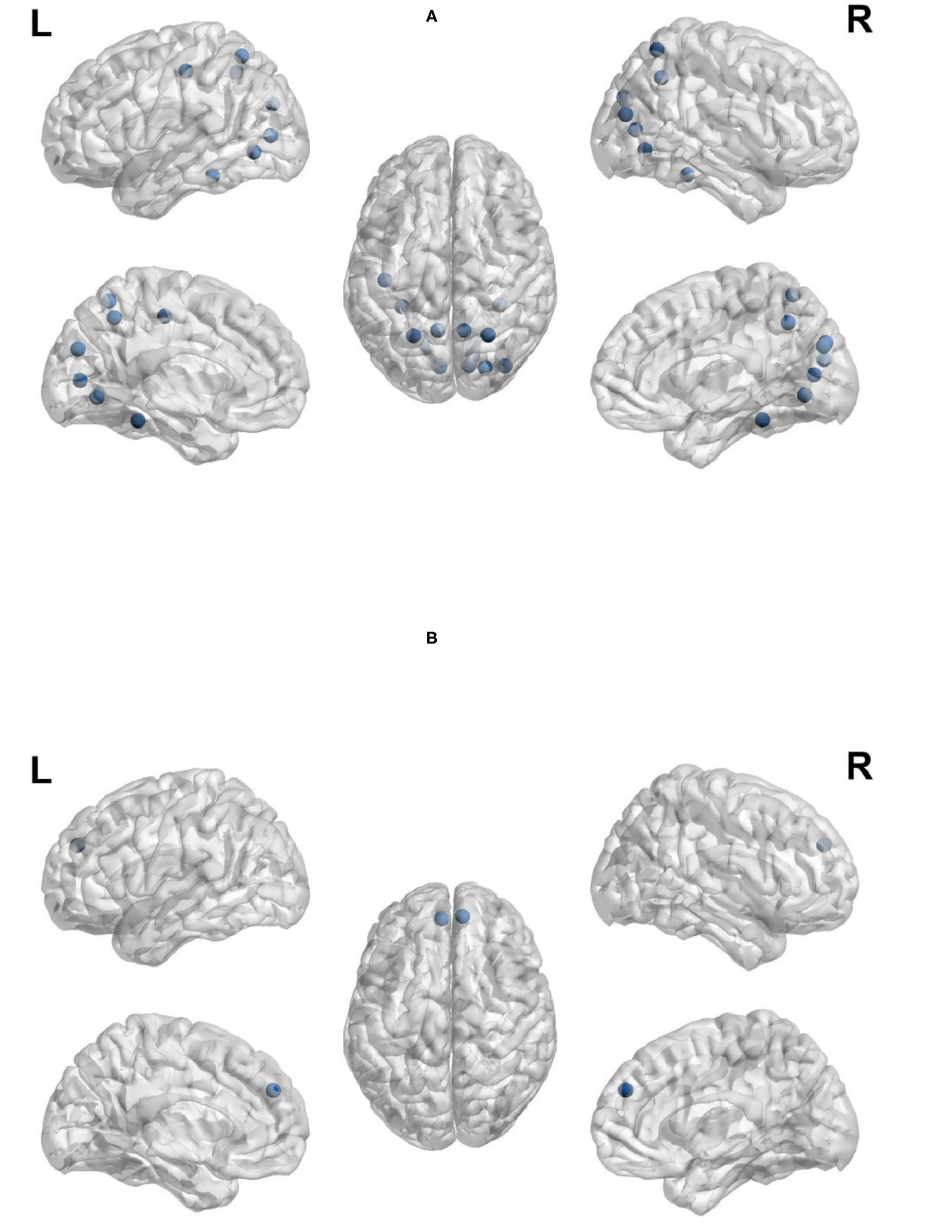
Anatomical regions with higher node betweenness during planning (Top) and during execution (Bottom). **(A)** Anatomical regions with higher node betweenness during planning. **(B)** Anatomical regions with higher node betweenness during exection.

Node out-strength is a measure of how connected the node is to other nodes in the network. Figures 9A,B visualize the brain regions with higher node out-strength during planning and during execution, respectively. Planning showed greater out-strength than execution in the following regions: occipital regions (calcarine, cuneus), parietal regions (bilateral superior parietal cortex and precunues), the right superior frontal cortex, and inferior occipito-temporal regions (fusiform and lingual gyri). The left angular gyrus and bilateral medial superior frontal cortex showed greater out-strength for execution. As for node betweenness, Figures 10A,B visualize the brain regions with higher betweenness during planning and during execution, respectively. The following brain regions had higher node betweenness during planning than execution: occipital regions (calcarine, cuneus, right middle, right superior); inferior occipito-temporal (fusiform, lingual); parietal (bilateral superior parietal, left postcentral, precuneus). Bilateral medial superior frontal had higher node betweenness during execution than planning.

These results suggest that there is greater information flow during planning than execution. This matches our expectations. Planning is more computationally demanding than execution. Again, during planning, participants must explore the problem space, which requires generating and manipulating a mental representation of the problem. The regions that show greater information flow during planning are all regions involved in that generation and manipulation, particularly parietal, occipital and inferior occipito-temporal. On the other hand, execution requires recall of the plan generated and stored and therefore, greater information flow from frontal regions related to memory retrieval is observed.

**Figure 11:**
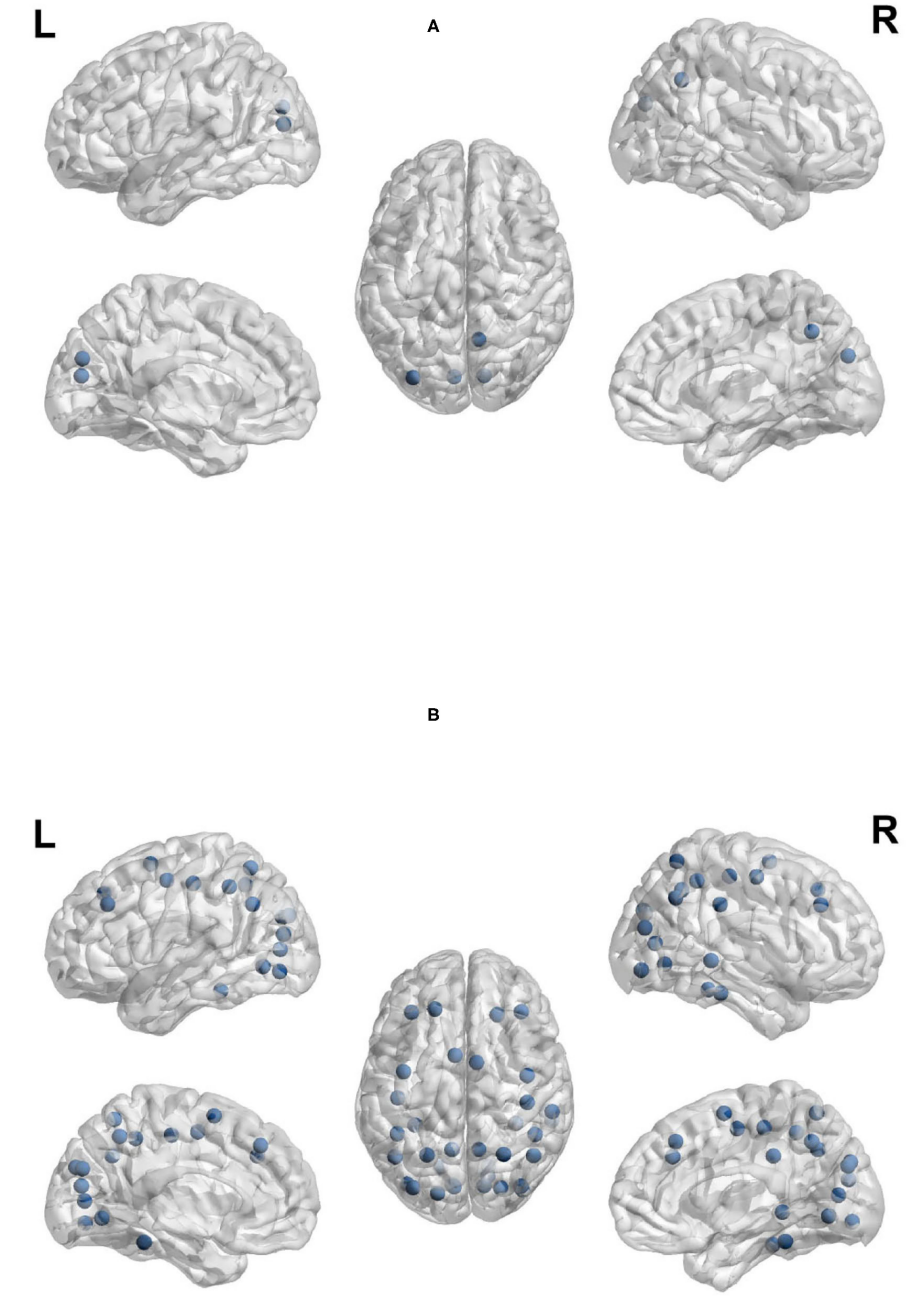
Anatomical regions with higher local efficiency and clustering coefficient (Top) and higher transitivity (Bottom) during planning. **(A)** Anatomical regions with higher local efficiency and clustering coefficient during planning. **(B)** Anatomical regions with higher transitivity during planning.

Clustering coefficient, local efficiency and transitivity are measures of segregation that aim to identify sub-networks. Figure 11A visualizes the brain regions with higher local efficiency and higher clustering coefficient during planning phase compared to execution phase. While Figure 11B visualizes the brain regions with higher transitivity during planning than during execution phase. Each of these measures was larger for planning than execution, with no regions showing larger measures for execution.

The regions that showed a higher clustering coefficient in planning included: the cuneus, left middle occipital cortex, and right precuneus. Local efficiency was higher in a similar set of regions (the cuneus, left middle occipital cortex, and right precuneus). The clustering coefficient and local efficiency identified a visual-spatial sub-network that is more strongly connected during planning. Transitivity identified an overlapping but more extensive set of regions that included: bilateral angular gyrus, calcarine sulcus, cuneus, bilateral middle frontal cortex, bilaterial superior frontal cortex, bilateral fusiform and lingual gyri, bilateral occipital cortex, bilatral superior parietal cortex, postcentral and precentral cortex, precuneus, supplementary motor area, right supramarginal gyrus, and right inferior and middle temporal cortex.

### 5.3. Global Efficiency

Since global efficiency is measured over the entire brain network, not for a given node in the network, we measured the global efficiency for all planning and execution networks within all runs across subjects. Then, global efficiency of planning is compared against that of execution. Results show that the majority of runs had higher global efficiency scores during planning than execution; 43 out of 72 runs had higher global efficiency during planning than execution. Furthermore, Table 6 shows the number of runs where global efficiency was higher during planning and during execution across all subjects for all 4 runs of each subject. The first column shows the number of subjects that had a higher global efficiency score during planning than during execution. The second column shows the number of subjects that had a higher global efficiency score during execution than during planning.

**Table 6:** Global efficiency.

**Run number**	**Planning**	**Execution**
1	15	3
2	9	9
3	10	8
4	9	9

Although there was no significant difference in global efficiency between planning and execution, from the table, it is clear that the majority of subjects had a higher global efficiency for planning for the first runs. Some subjects switched from having higher global efficiency during planning to having higher global efficiency during execution. A potential explanation for this change across runs is switching from pre-planning to online planning or planning intermixed with execution. Although there is a dedicated planning phase in the current study, that does not mean that planning is not taking place during execution. In fact, it has been debated as to whether efficient pre-planning is possible in the TOL or whether TOL performance is controlled by online planning (Kafer and Hunter, 1997; Phillips, 1999, 2001; Unterrainer et al., 2004). According to Phillips (1999, 2001), pre-planning the entire sequence is not natural, but that people instead plan the beginning sequence of moves and then intersperse planning and execution. If this is the case, then it may be expected that some participants will switch to online planning. This intermixing of planning and execution is also likely to impact the performance of the machine learning algorithms to detect planning and execution phases.

The relationship between global efficiency and behavioral performance was examined. Global efficiency was found to be positively correlated with the mean number of extra moves (a measure of error) during problem-solving (for execution *r* = 0.73, *p* = 0.0006). Previous studies have shown a relationship between global efficiency and task performance (Stanley et al., 2015).

This suggests that the variance in global efficiency is indicative of individual differences in neural processing and further suggests that the changes in global efficiency across runs are also likely indicative of changes in neural processing related to changing strategy. Further research using a larger sample is necessary to explore this hypothesis.

## 6. Conclusion

In this paper, we propose a new computational method to estimate dynamic functional brain networks from the fMRI signal recorded during a complex problem solving task. Our model recognizes the two phases of complex problem solving with more than 80% accuracy, indicating the representation power of the suggested dynamic brain network model. We study the properties of the constructed brain networks during planning and execution phases in order to identify essential anatomic regions in the brain networks related to problem solving. We investigate the potential hubs and densely connected clusters. Furthermore, we compare the network structure of the estimated dynamic brain networks for planning and execution tasks.

There are some limitations to the study. Although the primary aim of this study was to demonstrate the feasibility of the methods, the sample size is somewhat small, making the interpretation of the results difficult. Second, a goal of this method is to identify brain states that are interspersed with each other. In the current study, planning was expected to occur both prior to execution as well as during execution; therefore, planning states are interspersed within the execution phase. The temporal sampling rate of the fMRI data may be a limiting factor. Alternatively, the sluggish and blurred underlying hemodynamic response may be the factor preventing the ability to detect brain states. We plan to explore this factor in future work.

## Data Availability Statement

The dataset used in this study and the code required to reproduce our results can be found at https://osf.io/krch2/?view_only=df8aaf1f4fa46129a69f89486f65a83.

## Ethics Statement

The studies involving human participants were reviewed and approved by Indiana University Institutional Review Board. The patients/participants provided their written informed consent to participate in this study. All procedures performed in studies involving human participants were in accordance with the ethical standards of the institutional and/or national research committee and with the 1964 Helsinki declaration and its later amendments or comparable ethical standards. All individual participants in the study signed an informed, written consent documents approved by the Indiana University Institutional Review Board.

## Author Contributions

AA proposed and implemented the computational model with support from OE and BK. Performed the experiments with support from BK and OE. Prepared the visualizations with support from BK. Wrote the manuscript with support from OE, SN, and FY. BK proposed the idea for building brain networks using neural networks. SN provided the neuroscientific interpretations of the results of the proposed computational model, designed the Tower of London experiment procedure and collected the corresponding fMRI recordings. FY supervised the project, conceived the study and was in charge of the overall direction and planning. All authors provided critical feedback and helped shape the research, analysis and manuscript. All authors contributed to the article and approved the submitted version.

## Funding

This study was funded by TUBITAK (Scientific and Technological Research Council of Turkey) under grant No: 116E091 as well as the Indiana METACyt Initiative of Indiana University, funded in part through a major grant from the Lilly Endowment, Inc. AA, OE, BK, and FY received funding from TUBITAK (Scientific and Technological Research Council of Turkey) under the grant no: 116E091. SN received research funding from the Indiana METACyt Initiative of Indiana University and a major grant from the Lilly Endowment, Inc. The authors declare that this study received funding from the Lilly Endowment Inc. The funder was not involved in the study design, collection, analysis, interpretation of data, the writing of this article or the decision to submit it for publication.

## Conflict of Interest

The authors declare that the research was conducted in the absence of any commercial or financial relationships that could be construed as a potential conflict of interest.

## Publisher's Note

All claims expressed in this article are solely those of the authors and do not necessarily represent those of their affiliated organizations, or those of the publisher, the editors and the reviewers. Any product that may be evaluated in this article, or claim that may be made by its manufacturer, is not guaranteed or endorsed by the publisher.
